# Lymph Leakage Promotes Immunosuppression by Enhancing Anti-Inflammatory Macrophage Polarization

**DOI:** 10.3389/fimmu.2022.841641

**Published:** 2022-05-19

**Authors:** Andrés A. Herrada, Alexandra Olate-Briones, Rodrigo Lazo-Amador, Chaohong Liu, Bairon Hernández-Rojas, Gonzalo Riadi, Noelia Escobedo

**Affiliations:** ^1^ Lymphatic Vasculature and Inflammation Research Laboratory, Instituto de Ciencias Biomédicas, Facultad de Ciencias de la Salud, Universidad Autónoma de Chile, Talca, Chile; ^2^ Department of Pathogen Biology, School of Basic Medicine, Tongji Medical College, Huazhong University of Science and Technology, Wuhan, China; ^3^ Ph.D Program in Sciences Mention in Modeling of Chemical and Biological Systems, Faculty of Engineering, University of Talca, Talca, Chile; ^4^ Agencia Nacional de Investigación y Desarrollo (ANID) – Millennium Science Initiative Program Millennium Nucleus of Ion Channels-Associated Diseases (MiNICAD), Center for Bioinformatics, Simulation and Modeling, CBSM, Department of Bioinformatics, Faculty of Engineering, University of Talca, Talca, Chile

**Keywords:** lymphatic vasculature, macrophages, inflammation, cancer, inflammatory bowel disease

## Abstract

Lymphatic vasculature is a network of capillaries and vessels capable of draining extracellular fluid back to blood circulation and to facilitate immune cell migration. Although the role of the lymphatic vasculature as coordinator of fluid homeostasis has been extensively studied, the consequences of abnormal lymphatic vasculature function and impaired lymph drainage have been mostly unexplored. Here, by using the *Prox1^+/–^* mice with defective lymphatic vasculature and lymphatic leakage, we provide evidence showing that lymph leakage induces an immunosuppressive environment by promoting anti-inflammatory M2 macrophage polarization in different inflammatory conditions. In fact, by using a mouse model of tail lymphedema where lymphatic vessels are thermal ablated leading to lymph accumulation, an increasing number of anti-inflammatory M2 macrophages are found in the lymphedematous tissue. Moreover, RNA-seq analysis from different human tumors shows that reduced lymphatic signature, a hallmark of lymphatic dysfunction, is associated with increased M2 and reduced M1 macrophage signatures, impacting the survival of the patients. In summary, we show that lymphatic vascular leakage promotes an immunosuppressive environment by enhancing anti-inflammatory macrophage differentiation, with relevance in clinical conditions such as inflammatory bowel diseases or cancer.

## Introduction

The lymphatic vasculature (LV) is a one-way network of thin-walled capillaries and larger vessels enveloped by a continuous layer of endothelial cells. Its principal function is to maintain fluid homeostasis by draining the protein-rich lymph, small molecules, and cells (collectively termed lymph) from the tissue extracellular spaces and returning them to the venous system (1). In addition to this main function, LV is also involved in the absorption of dietary lipids in the gastrointestinal organs and the management and coordination of immune cell trafficking into lymph nodes and inflammation (2–4). Moreover, in recent years, LV malfunction has been associated with various pathologies such as inflammatory bowel disease (IBD) (5), obesity (6, 7), glaucoma (8), Alzheimer’s disease (9) and lymphedema (10, 11) among others. In most of these pathologies, LV malfunction leads to lymph accumulation, reduced protein clearance and inflammation (8, 10–13). Despite the fact that the role of malfunction LV in promoting different diseases has begun to be clarified, the consequences of lymph accumulation in tissues still remain vastly elusive (7). In fact, by using the *Prox1^+/–^* mice with defective lymphatic vasculature and lymphatic leakage, we and others have previously shown that these animals develop features characteristic of late-onset obesity resulting from subcutaneous and intra-abdominal fat accumulation, due to the subtle leakage of lymph fluid from defective lymphatics (6, 7, 14). The lymphatic vessels that were most severely affected in *Prox1^+/-^* mice were those from the viscera, particularly of the mesentery and intestine; ingestion of a fluorescent lipid concluded that these mesenteric vessels were leaky (6). Accordingly, lymph fluid promotes adipogenesis *in vitro*, suggesting that the direct contact of leaky lymph with the edematous tissue can promote adipogenesis (6). Although some data suggest that impaired lymph drainage can also affect immune and inflammatory responses, the direct consequences of lymph fluid accumulation over immune cell response have not been yet addressed (15).

Macrophages play a critical role in the initiation and maintenance of inflammation in response to infectious challenge or injury, starting the innate immune response and directing the adaptive immune response (16, 17). Macrophages exhibit polarized phenotypes that are broadly divided into two categories. Classically activated macrophages or M1, play essential roles in response to bacterial challenge and inflammation, whereas alternatively activated macrophages or M2, participate in debris scavenging and tissue remodeling, playing a key role in the resolution of inflammation (18). Imbalance of M1/M2 polarization can lead to inefficient immune responses and it has been involved in various pathological processes (19–21). In the tumor microenvironment for example, local mediators can modulate tumor-associated macrophages (TAMs) to polarize to a M2-like phenotype, dampening the overall immune response and promoting tumor immune surveillance escape (22). On the other hand, increased M2 macrophages seem to have an antifibrotic role in a mouse model of lymphedema (23). Thus, it is now clear that M1/M2 polarization is a dynamic process and involves the tissue microenvironment (24). Therefore, although great efforts have been directed to evaluate how different cytokines and metabolic pathways are involved in M1/M2 polarization, extrinsic signals that regulate macrophage status remain mostly unclear (25–28).

Here we show that lymph fluid can modulate the immune response by skewing macrophage polarization towards an M2 phenotype. Accordingly, *Prox1^+/–^* mice were protected from DSS-induced colitis and microscopic colon analysis showed increased M2 macrophage infiltration. On the other hand, *Prox1^+/–^* mice developed bigger tumors compared to littermate controls, with increased intratumoral M2 macrophage infiltration. In fact, RNA-seq analysis from human tumors revealed a reduced lymphatics gene signature, which is associated with reduced M1 and increased M2 macrophages signature in the tumor microenvironment, affecting patient survival. Furthermore, chyle from *Prox1^+/-^* mice, a milky bodily fluid consisting of lymph and emulsified fats which drains from the intestine into the lymphatic vessels during digestion, was also able to promote M2 polarization of primary macrophages *in vitro*. Our results highlight the importance of LV in modulating the immune response by regulating the M1/M2 macrophage ratio. Moreover, targeting malfunctioned LV may be used as a therapeutic strategy to promote a potent immune response to be used in different clinical conditions such as cancer.

## Materials and Methods

### Mice

Male and female *Prox1^+/–^* (*Prox1^+/LacZ^*), Prox1-tdTomato and *WT* mice (NMRI background) were kindly donated by Dr. Guillermo Oliver (Northwestern University, USA). Six- to eight-week-old mice were used in this study. Animals were housed in temperature- and humidity-controlled rooms, maintained on a 12-h light/12-h dark cycle (lights on at 7:00 hours). All animal procedures and experiments were performed according to animal protocols approved by the Institutional Animal Care and Use Committee at Universidad Autónoma de Chile.

### Dextran Sodium Sulfate (DSS) Colitis Model

Colitis was induced in mice by adding DSS (3% w/v) (MP Biomedicals) to drinking water for 8 days. Control groups (without DSS) only received drinking water. The general condition and health of the mice was monitored by a routine measurement of body weight and periodic observation. Animals were sacrificed by transcardial perfusion with phosphate-buffered saline 1X (PBS) at different time points (days 2, 5 and 12) and the small intestine and colon were carefully harvested. All tissues were measured, photographed and analyzed as described below.

### Colon Explant Cultures

Colons were isolated from mice, opened longitudinally, washed thoroughly in PBS with 100 U/ml penicillin G and 100 µg/ml streptomycin (Gibco), weighed, and ~0.5 cm sections were cultured in 500 µl RPMI-1640 (supplemented with 10% fetal bovine serum penicillin, streptomycin, and gentamicin) for 24 h at 37°C. Supernatants were then collected and analyzed by ELISA.

### Immunohistochemistry

Freshly isolated small intestine and colons were washed thoroughly with cold PBS and fixed in 4% ice-cold paraformaldehyde overnight (Sigma-Aldrich, St. Louis, MO). Fixed samples were then washed in PBS and dehydrated on a sucrose gradient with 15% sucrose in PBS, followed by 30% sucrose overnight. Next day, tissues were embedded in Tissue-Tek OCT (Sakura Finetek, Torrance, CA) and stored at -20°C before sectioning. For tail analysis, mice were euthanized, and their tails were obtained, sliced, fixed in 4% ice cold paraformaldehyde overnight (Sigma-Aldrich, St. Louis, MO), and decalcified using 3% EDTA (Loba Chemie Pvt Ltd., Mumbai, India) for 3 weeks, under gentle agitation on a shaker. Fixed samples were then washed in PBS and treated with 15% sucrose in PBS for 5 hours, followed by 30% sucrose overnight. Next day, tissues were embedded in Tissue-Tek OCT (Sakura Finetek, Torrance, CA) and stored at -20°C before sectioning. Samples were sectioned at 30 µm with a cryostat and processes for immunofluorescence. Briefly, samples were slightly permeabilized in PBS +0.5% Triton X-100 for 20 minutes, blocked with a mix of 5% donkey serum (Jackson ImmunoResearch) +1% bovine serum albumin +0.05% sodium azide and 0.1% Triton X-100 in PBS, and incubated overnight at room temperature with anti-LYVE-1 (Cat No. AF2125, RyD Systems) at 1:1000 dilution. The next day, samples were washed with PBS + 0.1% Triton X-100, followed by secondary antibody conjugated with Alexa 488 (Molecular Probes, #A21208). Immune cell infiltration was evaluated by using a mAb against F4/80 (Cat No. 35-4801-U100, TONBO) and CD206 (Cat No. 141712, BioLegend) both at 1:250 dilution, followed by a secondary antibody conjugated with Alexa 488 (Cat No. A21208, Molecular Probes). Samples were washed with PBS + 0.1% Triton X-100; mounted with VECTASHIELD antifade mounting medium with DAPI (Vector Laboratories) and sealed with nail polish. Images were acquired either in an Olympus BX51 epifluorescence Microscope (Olympus, Melville, NY) or in a Leica Stellaris 5 Confocal microscope (Leica Microsystems, Wetzlar, Germany). Lymphatic vascular area and cell count were manually quantified in a blinded manner using ImageJ software. The number of F4/80^+^ and CD206^+^ cells were quantified from the photos obtained in the confocal microscope at a magnitude of 60X after performing a z-stack and a maximum projection of the full stack. Cells were quantified by field and including photographs throughout all the cell layers of the colon (mucosa, submucosa and muscularis).

### ELISA

Serum samples were obtained the day before DSS treatment (day -1) and after DSS treatment (days 2, 5 and 12). For IL-6 detection, capture mAb (Cat No. 504502, BioLegend) and detection mAb (Cat No. 504602, BioLegend) were used following manufacturer’s protocol. IL-1β levels were measured by using antibodies specific for the mouse cytokine (capture mAb: Cat No. 503502 and detection mAb: Cat No. 515801, BioLegend), following manufacturer’s protocol. Samples were measured on an Autobio PHOmo microplate reader in a blinded manner.

### Real-Time Semiquantitative Polymerase Chain Reaction Analysis

Total RNA was isolated from the distal colon using TRIzol reagent according to manufacturer’s instructions (Thermo Fisher Scientific). RNA (1 µg) was reverse transcribed using the High-Capacity RNA to-cDNA Kit (Applied Biosystems) according to the manufacturer’s instructions. Quantitative reverse transcription PCR was performed using SYBR Green Real-Time PCR Master Mix (Thermo Fisher Scientific). The PCR primers used in this study were: IL-6 (forward, 5’-CTGCAAGAGA CTTCCATCCAGTT-3’, reverse 5’-GAAGTAGGGAAGGCCGTGG-3’), IL-1b (forward, 5’-GCAACTGTTCCTGAACTCAACT-3’, reverse 5’-ATCTTTTGGGGTCCGTCAACT-3’), and GAPDH (forward, 5`-GAGGCCGGTGCTGAGTATGT-3’, reverse 5’-GGTGGCAGTGATGGCATGGA-3’). Each complementary DNA sample was analyzed in duplicate or triplicate for quantitative assessment of RNA amplification, and the results are expressed relative to the housekeeping gene GAPDH.

### Macrophage Preparation and Polarization

The chyle was obtained directly from the abdominal cavity of *Prox1^+/-^* pups that present visible accumulation of chyle in the first 2-5 days after birth. Chyle was collected from the abdominal cavity of newborn *Prox1^+/-^* pups immediately after euthanasia. The chyle was centrifuged at 300 g for 5 mins and the supernatant was stored at -20°C until use. All the aliquots of chyle obtained from several *Prox1^+/-^* pups were pooled into a single stock and filtered on a 0.2 μm filter. 2 μl of this pooled chyle were used for the *in vitro* assays (described below). Peritoneal macrophages were obtained as described before(29). Briefly, cells from the abdominal cavity of mice were harvested by repeated lavage with 7 mL cold PBS/FBS 3%, using a 21-G needle. Cell viability was evaluated by the trypan blue dye exclusion method. Then, peritoneal cells were cultured in DMEM alone for 90 min, extensively washed with pre-warmed DMEM medium, and then enriched macrophages were cultured in DMEM medium with 10% FBS, supplemented with penicillin/streptomycin and glutamine overnight. The next day, macrophages were polarized to M0, (complete medium alone), M1 (100 ng/mL IFNγ and 20 ng/mL LPS), or M2 (20 ng/ml IL-4) with or without chyle or vehicle (2 µL) for 24 and 48 hours. Macrophage polarization was assessed by flow cytometry. M1 macrophages were defined as F4/80^+^CD11b^+^CD11c^+^CD206^-^ or F4/80^+^CD11b^+^ CD11c^+^ CD301^-^ and M2 macrophages were defined as F4/80^+^CD11b^+^CD11c^-^CD206^+^ or F4/80^+^ CD11b^+^CD11c^-^CD301^+^ as previously described (30, 31). Bone marrow cells were isolated from 6- to 8-week-old wild-type C57BL/6 mice. 2.5 × 10^5^ cells per well were cultured for 7 days in DMEM supplemented with 10% heated-inactivated FBS plus penicillin/streptomycin in the presence of 10 ng of M-CSF. After 7 days, cultures contained >97% macrophages as assessed by CD11b and F4/80 staining. Polarization was performed as described above in the presence of chyle or vehicle and analyzed 24 hours later.

### Isolation of the Stromal Vascular Fraction (SVF) Cells

White adipose tissue from the abdominal cavity was obtained as previously described (32). Briefly, adipose depots were isolated, rinsed and minced in KRB solution (HBSS diluted 1x in PBS; 2% BSA, 12.5 mM HEPES) with 1 mg/ml Collagenase II (Sigma) and 0.2 mg/ml DNAse I (Sigma) at 37°C with high-speed shaking. Cell suspension was filtered through a 100 µM nylon sieve and centrifuged at 500×g at room temperature for 5 minutes. Red blood cells were lysed with erythrocyte lysis (ACK) buffer, centrifuged and pellet SVF cells were staining for FACS analysis.

### Mouse Model of Tail Lymphedema

Acquired lymphedema was surgically induced in the tails of C57BL/6 mice as previously described (33, 34). Briefly, a full-thickness circumferential incision of the skin was made 15 mm distal to the base of the mouse tail. Lymphatic trunks through controlled limited cautery application (lymphedema) or just skin incision without lymphatic cautery (Sham) were performed. Mice were daily monitored to record the lymphedema progression.

### Tumor Model

Tumors were induced by subcutaneous injection of 3x10^5^ B16/F10 cells on the back of *Prox1^+/-^* and littermate control mice. Tumor growth was recorded every two days with a caliper and calculated as V = 0.52 x d2 x D (D, long diameter; d, short diameter). After 3 weeks, the mice were sacrificed, and the tumors were excised for further analysis.

### Flow Cytometry

For macrophage polarization, cells were stained with a FITC-conjugated anti-F4/80 (Cat No. 35-4801-U500, TONBO), PerCP-conjugated anti-CD11b (Cat No. 65-0112-U100, TONBO), PE-conjugated anti-CD301 (Cat No. 145704, BioLegend), APC-conjugated anti-CD11c (Cat No. 20-0114-U100, TONBO), PE-conjugated anti-CD11 (Cat No. 12-0114-82, eBioscience) and Alexa Fluor-647-conjugated anti-CD206 (Cat No. 141712, BioLegend). Intratumoral M2 macrophages were determined by staining tumor cells with a FITC-conjugated anti-F4/80 and Alexa Fluor-647-conjugated anti-CD206. Samples were analyzed by flow cytometry using a FACS Calibur instrument and BD FACSDiva software (version 6.1.1; BD Biosciences, San Jose, CA, USA).

### Gene Signatures

The expression of the gene signatures (**Supplementary Table 1**), between Macrophages (M1 or M2) and LV signatures were correlated. Furthermore, Kaplan-Meier curves (K-M curves) for each gene signature were generated. For additional methods, see **Supplementary Information**.

### Statistical Analysis

Statistical analysis was performed using Prism (GraphPad Software, USA). The data are expressed as the means ± standard deviation of the mean (SD) or standard error (SEM). Data distributions were tested for normality using Shapiro-Wilk normality test (https://www.statskingdom.com/320ShapiroWilk.html). The significant differences between different data were calculated by unpaired two-tailed t-test (for two groups) and one-way ANOVA (for more than two groups), followed by Tukey’s multiple comparison test. All P values < 0.05 were considered significant. *: P < 0.05; **: P < 0.01; ***: P < 0.001; ****: P < 0.0001.

## Results

### Lymphatic Dysfunction Protects Animals From DSS-Induced Colitis

**Figure 1:**
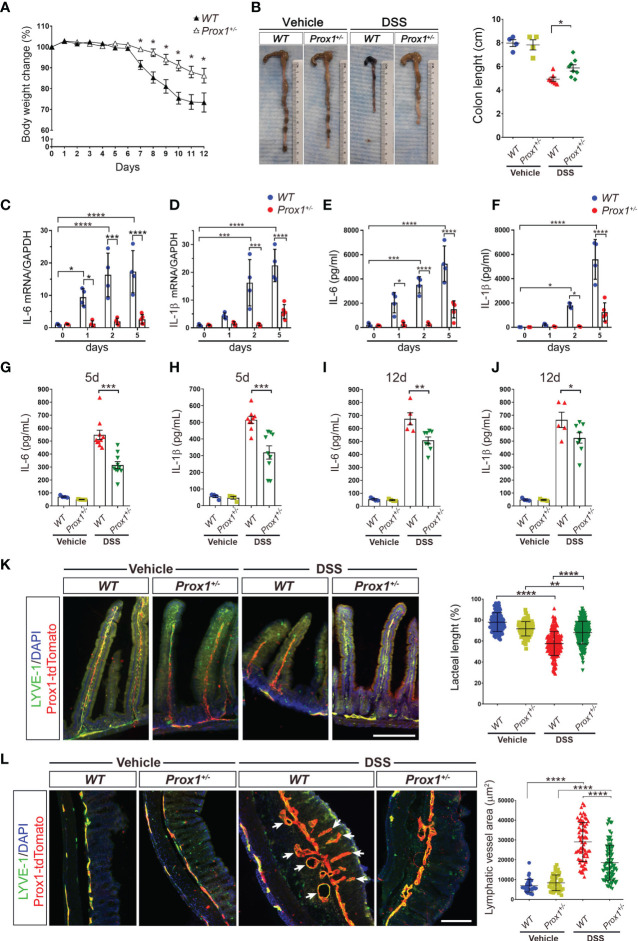
*Prox1^+/-^* mice are protected from DSS-induced colitis. **(A)** Mouse body weight changes during DSS treatment. *Prox1^+/-^* and NMRI littermate controls (*WT*), were treated with 3% (w/v) DSS in the drinking water for 8 days and the weight was measured daily for 12 days. Body weight changes were calculated as a percentage of weight prior to DSS treatment (Day 0) Data are plotted as means ± SEM from male and female mice of at least 10 mice per genotype. Results are representative of 2 independent experiments. * P < 0.05, 1-way ANOVA followed by Tukey´s multiple comparison test. **(B)** Macroscopic appearance (left) and quantification of the length of colons (right) at day 12 from *WT* and *Prox1^+/-^* mice treated with DSS or vehicle. Data are plotted as means ± SEM. **(C, D)** mRNA levels of IL-6 **(C)** and IL-1β **(D)** in colon samples of *WT* and *Prox1^+/-^* mice treated with DSS. **(E, F)** Time course of cytokine levels in in colonic tissue explants showing the effect of DSS treatment in *WT* and *Prox1^+/-^* mice. Tissue levels of IL-16 **(E)** and IL-1β **(F)** in colon were quantified by ELISA assay. Data expressed as mean ± SD and statistical significance evaluated by One-way ANOVA followed by Tukey’s post-test where * P < 0.05; *** P < 0.001; **** P < 0.0001. **(G–J)** Serum levels of IL-6 and IL-1β from the different groups evaluated at day 5 **(C, D)** and day 12 **(E, F)** after DSS or vehicle treatment was determined by ELISA. Data are plotted as means ± SEM. * P < 0.05; ** P < 0.01; *** P < 0.001; 1-way ANOVA followed by Tukey´s multiple comparison test. **(K)** Lacteal length shrinking is reduced in DSS-treated *Prox1^+/-^* mice compared to DSS-treated *WT* animals. (Left) Representative images of lacteals (LYVE-1, green; Prox1-tdTomato, red) on thick sections of the small intestine (proximal duodenum) from *WT* and *Prox1^+/-^* mice treated with vehicle or DSS for 12 days are shown. Scale bar is 200 µm (Right). Quantification of relative lacteal length from the different groups is shown. Dots indicate values of 113–225 villi/group in n = 5 mice/group pooled from two independent experiments. Data are plotted as means ± SD. ** P < 0.01; **** P < 0.0001; **(L)** Reduced colonic lymphatic vasculature area in *Prox1^+/-^* mice treated with DSS. The colon lymphatics were stained using LYVE-1 antibody (green) and using the transgenic Prox1-tdTomato mice (red). LYVE-1+ Prox1+ indicates the lymphatic vessels (left). Scale bar is 200 µm. Quantification of the area of colon lymphatics vessels (right). Dots indicate values of 34–103 villi/group in n = 5 mice/group pooled from two independent experiments. Error bars indicate SD. **** P < 0.0001 by 1-way ANOVA with *post hoc* Tukey’s test.

LV remodeling and dysfunction, especially in initial lymphatics in the villi of the small intestine, called lacteals, has been observed in humans and animal models suffering from IBD (35, 36), although how these LV alterations impact disease progression is not fully understood. We first decided to evaluate LV remodeling during IBD by using the Dextran sodium sulfate (DSS) colitis model, that induces a strong intestinal inflammation, especially in the colon, with symptoms resembling those observed in human ulcerative colitis (37). The exact mechanism whereby DSS causes inflammation is not fully explained; however, it induces reproducible colitis in rodents, and it is an established model of IBD. Although DSS is reported to have its main site of action in the large intestine, it is also known to affect the small intestine (38, 39). DSS treatment in drinking water on Wild Type (*WT*) mice in NMRI background (herein *WT* mice), showed dramatic weight loss and colon length shortening, two classical symptoms of colitis (**Supplementary Figures 1A, B**). Lacteal remodeling and regression were evident after 9 days of DSS-treatment (**Supplementary Figure 1C**), consistent with previous studies (38). Since DSS treatment induces inflammation mainly in the colon, we decided to also evaluate lymphatic structure in this tissue, and we observed dramatic remodeling as early as 5 days after DSS-treatment (**Supplementary Figure 1D**). We next analyzed the impact of a defective LV over colitis development by using the *Prox1^+/–^* mouse model, characterized by dysfunctional lymphatic vessels including leakage of chyle present in the peritoneal cavity; reduced clearance and widespread lymphatic vascular leak (6, 14). Surprisingly, body weight loss and colon shortening were less severe in *Prox1^+/–^* mice compared to *WT* littermate controls (**Figures 1A, B**). To assess whether *Prox1^+/–^* mice have reduced colonic inflammation after DSS treatment, we evaluated the mRNA levels of two proinflammatory cytokines, IL-6 and IL-1β, at different days after treatment. The results showed significant increase in the mRNA levels of these two cytokines in the *WT* mice, indicating local inflammation, while *Prox1^+/–^* mice did not show local inflammation in response to DSS treatment (**Figures 1C, D**). To confirm these results, we directly measured these proinflammatory cytokines released from colonic explant cultures of *WT* and *Prox1^+/–^* animals harvested at different days after DSS treatment. The results showed reduced IL-6 and IL-1β production by colon explants from *Prox1^+/-^* mice compared to *WT* littermate controls after DSS treatment, suggesting a reduced colonic inflammation in this mouse model (**Figures 1E, F**). Moreover, serum levels of pro-inflammatory cytokines were reduced in LV-defective animals treated with DSS, suggesting that systemic inflammation is also reduced (**Figures 1G–J**). We then evaluated whether morphological changes occur in the small and large intestine during DSS treatment by staining LV. When we analyzed LV remodeling, we observed reduced LV morphological changes in both the small and large intestine in *Prox1^+/–^* mice compared to *WT* littermate controls (**Figures 1K, L**). The most striking finding in the small intestine was the reduced lacteal length in *WT* and *Prox1^+/-^* mice by DSS treatment; however, the *Prox1^+/-^* mouse is significantly more resistant to this morphological change compared to its *WT* littermate (**Figure 1K**). Moreover, a substantial increase in Lyve1/Prox1 staining area was observed in the large intestine of *WT* and *Prox1^+/-^* mice after DSS administration, indicating a proliferating lymphangiogenic response (**Figure 1L**). This growth of the LV area in response to inflammation is much more exacerbated in the *WT* mouse than in *Prox1^+/-^*. Even in the *WT* samples, swelling was observed in the mucosal and submucosal areas, which is accompanied by a large increase in LV density (**Figure 1L**, white arrows). This is not observed in the *Prox1^+/-^* mouse after treatment with DSS. Quantification of Lyve1^+^/Prox1^+^ staining area further confirmed that *Prox1^+/-^* mouse is significantly more resistant to inflammation with DSS compared to its *WT* littermate. Together, these results suggest that defective LVs protect animals from DSS-induced colitis and chronic inflammation.

### Increased M2 Macrophages Infiltration in Colon From DSS-Treated *Prox1^+/–^* Mice

**Figure 2:**
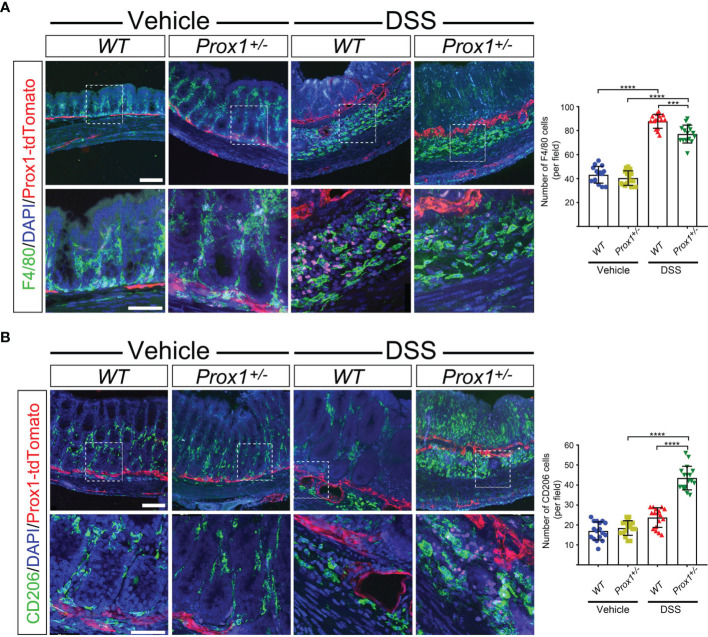
Increased M2 macrophages in colon from DSS-treated *Prox1^+/-^* mice. **(A, B)** Representative fluorescent immunohistochemistry of macrophages in colon from *WT* and *Prox1^+/-^* mice treated with vehicle (water) or DSS by 8 days followed by treatment with only water until day 12. Colon cryosections were immunostained with specific antibodies for F4/80 **(A)** or CD206 **(B)**. The images were acquired in a Confocal microscope at different magnifications: 20X (top panel) and 60X (bottom panel). The number positive cells were quantified from the 60X photographs after performing a z-stack and maximum projection covering the different layers of the colon sections (mucosa, submucosa and muscularis) (left panels). Scale bar is 100 µm (top panels in **A, B**) and 50 µm (bottom panels in **A, B**). Error bars indicate SD. ***P < 0.001; ****P < 0.0001 by 1-way ANOVA followed by Tukey´s multiple comparison test.

Because of our observation that *Prox1^+/–^* mice are protected from DSS-induced colitis, we next decided to evaluate the immune cell type responsible for this effect. We focused on the colon, since DSS induces inflammation specifically in this tissue. Thus, we evaluated macrophage infiltration by confocal microscopy. Our results showed that after DSS-treatment, an increased number of macrophages were observed in both *WT* and *Prox1^+/–^* mice, localized in the colon very close to lymphatics, suggesting cell infiltration (**Figures 2A, B**). When we dissected the type of infiltrating macrophage in the colon of DSS-treated mice, we found that *Prox1^+/–^* mice showed increased numbers of M2 macrophages close to lymphatics structures (**Figures 2A, B**, higher magnifications and quantifications), suggesting that this anti-inflammatory macrophage subpopulation could be playing a protective role in the DSS-colitis model.

### Lymph Promotes M2 Macrophage Polarization

**Figure 3:**
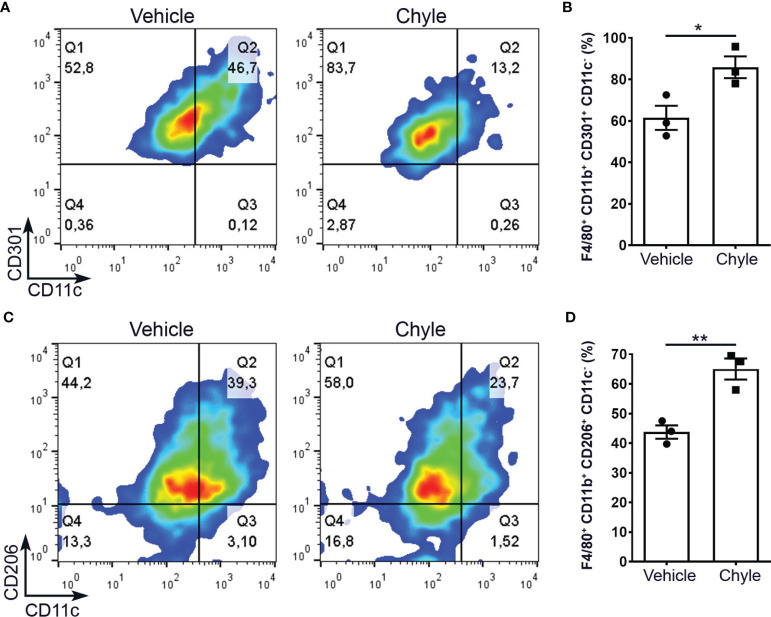
Chyle from *Prox1^+/-^* mice promotes M2 macrophage differentiation *in vitro*. Peritoneal macrophages were obtained from *WT* mice and cultured under M2 polarization condition with or without 2 µL of chyle or vehicle for 48 hrs. M2 macrophages were defined as F4/80^+^CD11b^+^CD301^+^CD11c^-^ **(A, B)** or F4/80^+^CD11b^+^CD206^+^CD11c^-^ **(C, D)** by FACS. Representative density plots (left) and quantification of 3 independent experiments (right) are shown. Error bars indicate SEM. *P < 0.05; **P < 0.01 by Unpaired t-test.

Since the increased amount of M2 macrophages close to lymphatics observed in the colon of DSS-treated *Prox1^+/–^* mice, and because *Prox1^+/–^* animals have LV dysfunction and lymph leakage, we decided to evaluate if chyle can help to polarize macrophages into M2 phenotype. Chyle corresponds to the lipid-rich fluid transported by lymphatic vessels of the intestine. Disruption and/or obstruction of the intestinal lymphatic vasculature results in chylous ascites, due to the leakage of this fluid into the abdominal cavity. While 70% of *Prox1^+/-^* embryos display edema and die at birth due to chylothorax and chylous ascites (6), we extracted chyle from the abdominal cavity of surviving *Prox1^+/-^* pups and used it in our *in vitro* study. It has been previously shown that lymph extracted from *WT* or *Prox1^+/–^* mice exert the same adipogenic effect *in vitro* (14). Additionally, *WT* and *Prox1^+/–^* lymph were analyzed by gas chromatography and mass spectrometry and no significant differences were found in their composition (14). Thus, peritoneal macrophages from *WT* mice were cultured under M2 conditional medium in the presence or absence of chyle from *Prox1^+/–^* pups. Our results showed stronger M2 polarization, defined as F4/80^+^CD11b^+^CD11c^-^CD206^+^ or F4/80^+^ CD11b^+^CD11c^-^CD301^+^, in macrophages cultured in the presence of chyle (**Figures 3A–D**). Similar results were obtained with bone-marrow derived macrophages (**Supplementary Figure 2**). Next, we decided to evaluate if chyle from *Prox1* ^+/-^ mice can also polarize macrophages towards a M2 phenotype *in vivo*. It has been previously described that leaky lymphatic in the *Prox1^+/-^* mice is more severe in the abdominal cavity and mesenteric vessels (6), so we analyzed the M1/M2 macrophages population in stromal vascular fraction isolated from abdominal adipose tissue from *WT* and *Prox1^+/–^* young animals. We observed an increased percentage of M2 macrophages in the stromal vascular fraction in mice with defective LV (*Prox1^+/-^*) compared to *WT* animals (**Supplementary Figure 3**), suggesting that the lymph that leaks out of the LV stimulates M2 macrophage polarization *in vivo*. To directly evaluate the role of the lymph in promoting M2 macrophage polarization *in vivo* in a different experimental model, we decided to use a mouse model of tail lymphedema, where thermal ablation of deep collecting lymphatic vessels leads to lymph accumulation and swelling of the tissue (40). In line with our previous results, we observed increased numbers of M2 macrophages in the lymphedematous tissue compared to the sham control group (**Supplementary Figure 4**), suggesting that lymph accumulation favors M2 macrophage polarization *in vivo* in this mouse model.

### Increased Tumor Growth and Tumor-Infiltrating M2 Macrophages in LV Defective Mice

**Figure 4:**
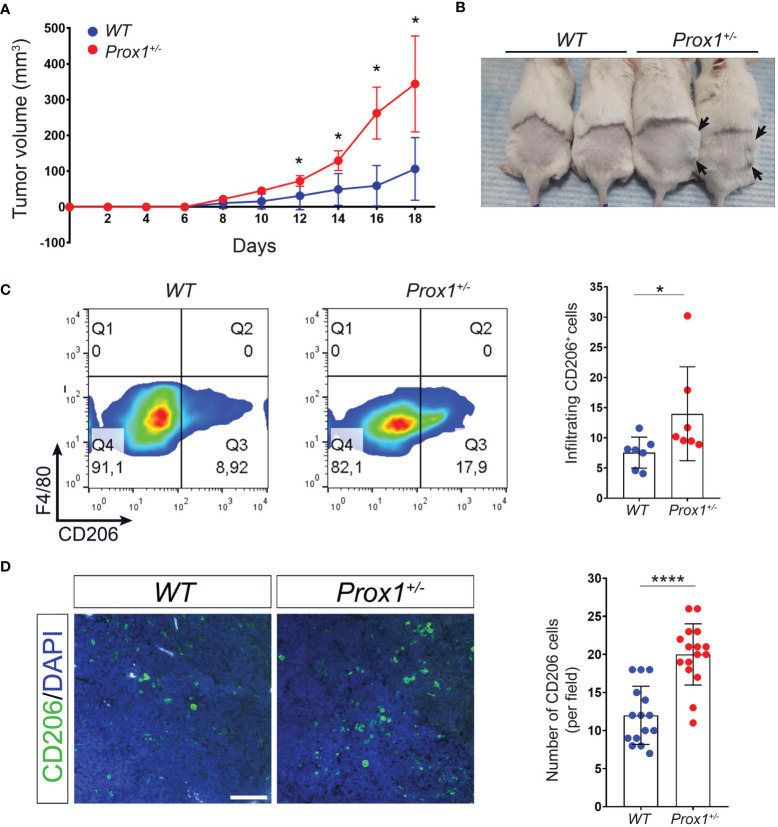
Defective lymphatics promote tumor growth and increased intratumoral M2 macrophages. B16F10 melanoma was injected in *Prox1^+/-^* and *WT* mice. Tumor volume was monitored every 48(h) **(A)** Growth curve of tumors in *Prox1^+/-^* and *WT* mice. **(B)** Representative tumors in *Prox1^+/-^* and *WT* mice on day 16 after tumor inoculation. *Prox1^+/-^* mice had larger tumor size (black arrows) compared to *WT* littermates. **(C)** Surface CD206 expression (left) and quantification (right) in *Prox1^+/-^* and *WT* TAMs by FACS. **(D)** CD206^+^ cells in the tumor of *Prox1^+/-^* and *WT* mice were analyzed by immunofluorescence staining. Representative images (left) and quantification (right) are shown. Scale bar is 100 µM. Data pooled from 3 (n = 12 mice per group) independent experiments is shown. Error bars indicate SEM. *P < 0.05; ****P < 0.0001 by 1-way ANOVA followed by Tukey´s multiple comparison test **(A)** or Unpaired t-test **(C, D)**.

We next evaluated in a different inflammatory context, the role of defective lymphatics in inflammation. Although LV has been implicated in tumor metastasis (41–43), less is known about the role of LV function and dysfunction in local tumor growth. Thus, tumors were induced by subcutaneous injection of B16/F10 cells, a murine melanoma cell line, on the back of *Prox1^+/–^* and *WT* littermate controls mice. Our results showed that *Prox1^+/–^* mice developed larger tumors than their *WT* littermate controls (**Figures 4A, B**). Moreover, tumor-infiltrating macrophages from *Prox1^+/–^* mice showed increased M2 macrophages proportion compared to *WT* littermate controls (**Figures 4C, D**). These results suggest that LV dysfunction and lymph leakage promotes a local anti-inflammatory environment by inducing M2 macrophage polarization that allows accelerated tumor growth.

### LV Function Affects M1 and M2 Programing and Impact Survival in Different Human Cancers

**Figure 5:**
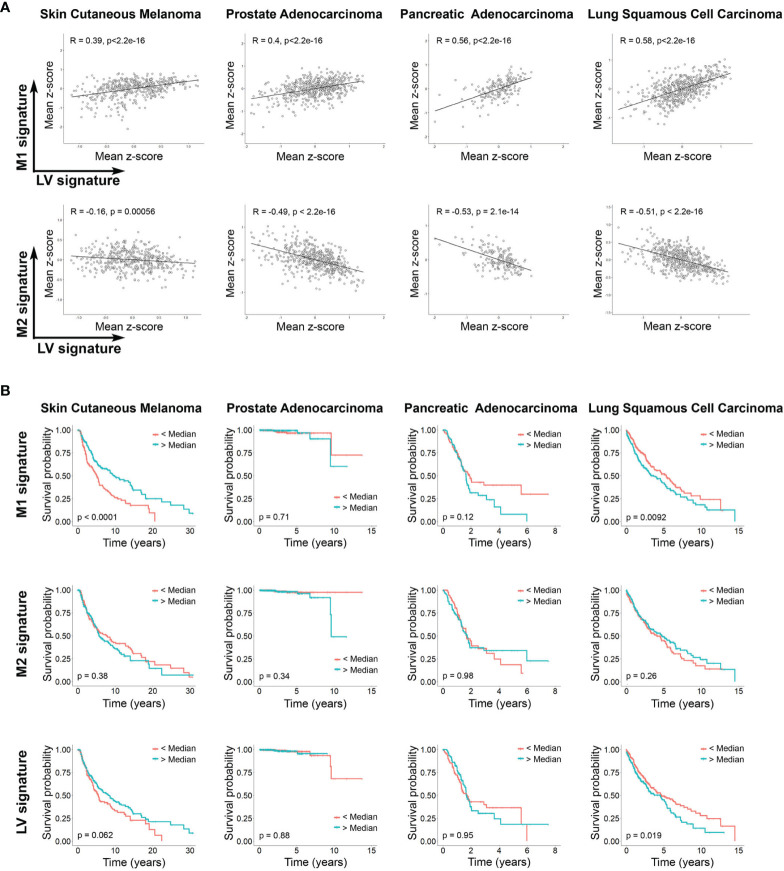
Lymphatic vasculature signaling differentially correlates with M1 and M2 cell signature and influences survival in different human cancers. **(A)** Correlation between signaling mean z-score gene signature of LV and M1, and LV and M2 (sample sizes: n_SKCM_ = 473; n_PAAD_ =183; n_PRAD_ = 550; n_LUSC_ = 553). Pearson correlation coefficients and P-values are shown. **(B)** Kaplan Meier plots showing the overall survival of patients (sample sizes: n_SKCM_ = 458; n_PAAD_ = 183; n_PRAD_ = 550; n_LUSC_ = 547 with patients’ available lifetime data) grouped according to the median z-score value of gene signatures (light blue curves: larger than median; red curves: smaller than median) corresponding to M1 and M2 cells and LV signaling. Resulting P-values from the log rank tests are shown per plot.

Finally, we set out to determine whether there is evidence of LV dysfunction and macrophage polarization in human cancers. By using previously described gene signatures for LV, M1 and M2 macrophages, and detailed in **Supplementary Table 1** (44, 45), we analyzed tumor transcriptomic data of patients with different cancers available within The Cancer Genome Atlas (46). Independent of the type of cancer evaluated, we found a striking positive correlation between LV and M1 macrophages signatures, and a negative correlation between LV and M2 macrophages signatures (**Figure 5A**). Since reduced LV signature can be related with reduced LV function and lymphedema (47–50), our finding is in keeping with our previous results that defective LV exacerbates M2 macrophage polarization in different inflammatory context and suggest a similar process in human cancers. Moreover, LV signature differentially correlates with patient’s survival depending on the cancer type analyzed and is also in line with the positive or negative association of M1 and M2 signature and cancer survival (**Figure 5B**). In fact, LYVE1, VEGF-C and ITGA9 are among the most down-regulated genes from the low LV signature patients among all the different cancer types analyzed (**Supplementary Figure 5**). All together, these results suggest that a similar dysfunctional LV modulates M1/M2 macrophage polarization within the tumor microenvironment in different human cancers.

## Discussion

Lymph leakage has been previously shown to promote *de novo* adipogenesis in the *Prox1^+/–^* mice, leading to obesity in adulthood (14). Whether lymph leakage affects other cellular processes is currently unknown. Here, we provide evidence suggesting that lymphatic dysfunction and lymphatic leakage can directly modulate the immune response, in part by perturbing the M1/M2 polarization status of macrophages in different inflammatory environments, impacting the outcome of different pathologies such as colitis and cancer.

During the last decades, increasing knowledge about the role of LV function in IBD has been documented, with observations showing that LV obstruction is common in Crohn’s disease patients together with dramatic changes in intestinal LV structures, changes that persist after inflammation resolve (38, 51, 52). Although it is still debated if these changes in LV morphology and function are a cause or consequence of IBD, the importance of LV function for inflammation resolution during IBD has been addressed by evidence showing that improving LV function ameliorates IBD symptoms (5), while blocking lymphangiogenesis exacerbates submucosal edema (53). However, no studies have shown the effect of a defective LV and lymph leakage before the development of IBD. Interestingly, intestinal lymphangiectasia is a rare congenital, acquired, or inherited disorder of the lymphatic vessels that involves lymphatic leakage into the intestine, somehow mimicking several of the characteristic seen in the intestine of *Prox1^+/-^* mice (54, 55). In these patients, it has been observed increased numbers of macrophages in the lamina propria with many of these macrophages acquiring a foam structure, although the phenotype of this population has not been evaluated (55–57). However, it has been previously shown that M2 macrophages are more likely to foam cell formation than pro-inflammatory M1 macrophages, suggesting that the macrophage accumulation observed in these patients could be potentially anti-inflammatory M2 macrophages (58). In fact, it has been observed in children with primary intestinal lymphangiectasia higher susceptibility to recurrent and opportunistic infections, suggesting a reduced immune response (59). Accordingly, lymphangiectasia is observed during DSS administration, and lymph leakage is reduced but does not disappear after recovery from intestinal inflammation, thus we can speculate that it can work as a safety control to maintain a M2 polarization condition (38). However, more research is necessary to directly evaluate anti-inflammatory macrophages in intestinal lymphangiectasia patients. Among the immune cells involved in promoting damage during IBD, macrophages play an essential role by either supporting or protecting damage according to their polarization status (60). Thus, while M2 macrophages protect against colitis by secreting immunosuppressive factors, M1 macrophages promote disease pathogenesis by secreting pro-inflammatory cytokines or directly attacking intestinal tissue (61–63). Accordingly, different molecules or treatments that increase M2 or reduce M1 polarization have been shown to protect from DSS-induced colitis (64). Leucrose, a natural sucrose isomer, suppresses DSS-induced colitis in mice by regulating macrophage polarization *via* JAK1/STAT6 signaling (65). Moreover, infection with helminth parasites has been shown to protect from DSS-induced colitis by promoting M2 macrophage polarization (66, 67). Interestingly, helminths can occupy and obstruct LV, which produces a chronic infection that causes lymphatic filariasis characterized by lymph leakage (68). Since helminth infection promotes macrophage polarization into a M2 phenotype (69), we can speculate that this effect can be in part explained by the LV damage and lymph leakage triggered by helminth infection. More research is necessary to confirm this idea.

Lymphedema is a painful, progressive condition generated by lymphatic dysfunction which leads to an accumulation of lymph, with swelling and fibrosis in the affected region, causing functional problems, chronic pain, and recurrent infection. Due to the high risk of infections in the compromised limb, persistent immunologic dysfunction may be present. Accordingly, altered humoral response, DC trafficking and elevated number of regulatory T cells (Tregs) have been described in patients and in immunized animal models (70–72). Moreover, an increased number of macrophages in the lymphedematous limb have been observed (73, 74). We have now shown in a mouse model of tail lymphedema increased numbers of M2 macrophages in the lymphedematous tissue at early days after lymphatic ablation, when lymph accumulation reaches a peak (33), suggesting that lymph could promote an anti-inflammatory macrophages polarization *in vivo*. These results are in line with a previous paper that showed increased number of M2 macrophages at later time points after ablation of lymphatic vessels (23). Thus, an overall immunologic dysfunction involving the presence of anti-inflammatory macrophages and Tregs, reduced DC trafficking and decreased humoral response may be responsible for the high risk of infections in these patients. If lymph is also able to promote the differentiation and function of Tregs remains to be evaluated.

The role of LV in cancer has been mainly focused on the metastasis process, where tumor cells use LV to spread into lymph nodes and then to other organs (75). However, it has been reported that LV also plays an important role in the tumor microenvironment, by studies showing that LV surrounding tumors have aberrant morphology, reduced function, and increased leakiness (76, 77). Malfunctioned LV promotes cancer growth in part by reducing lymphatic traffic of tumor-antigen-loaded antigen presenting cells (APC) into draining lymph nodes and thus reducing CD8^+^ T cell-mediated immune response (78). The results of another study indicated that dysfunctional peritumoral lymphatics caused edema, and reducing peritumoral lymphatic function by ablation of lymphatic vessels increased peritumoral edema, resulting in an accumulation of peritumoral Tregs, myeloid-derived suppressor cells, and rapid tumor growth in two murine tumor models, melanoma and breast cancer (79). Another study in a mouse model of melanoma showed that tumor-associated lymphatic endothelial cells (LECs) express Programmed death-ligand 1 (PD-L1), an immune checkpoint inhibitor, and in response to IFNγ production by antigen-specific CD8^+^ T cells, tumor-associated LECs increased PD-L1 expression limiting local effector CD8^+^ T cell accumulation (80). We have now shown here that chyle has anti-inflammatory properties, by modulating M1/M2 polarization process in macrophages, which could impact intratumoral immune responses and promote tumor growth. We can speculate that modulating tumoral LV either directly or indirectly by tumor cells could be a new strategy to dampen antitumoral immune responses. In fact, tumor cells are able to directly modulate LV function, by secreting the pro-lymphangiogenic vascular endothelial growth factor C (VEGF-C), promoting intratumoral lymphatic growth, dilating LV and impairing lymphatic pumping (81, 82). Interestingly, it has recently been shown that B16F10 tumor cell line overexpressing VEGF-C results in increased lymph leaked out of intratumoral LV very early during tumor growth, suggesting that VEGF-C secreted by tumoral cells not only has lymphangiogenic properties but also promotes lymph leakage (83). Thus, the role of LV during tumor growth seems to be a complex process, involving on one hand the expression of different proteins by LECs that dampen antigen-specific CD8^+^ T cell response and metastasis (80, 84), and on the other hand tumor cells actively secrete VEGF-C to modulate LV function, promoting lymph leakage that blocks APC traveling to draining lymph nodes in order to initiate a strong adaptive immune response, and promotes an intratumoral M2 polarization environment that reduces local anti-tumoral response, allowing tumor growth. Whether lymph leakage could affect the number or functional status of Tregs and myeloid-derived suppressor cells, or if targeting intratumoral LV can work as a therapeutic strategy to treat tumor growth remains to be evaluated.

It is well known that macrophages play a key role in the antitumoral immune response (85). Classically M1 macrophages are believed to play a role in antitumor immunity, while M2 macrophages promote immunosuppression and tumor escape (86). However, this concept has begun to be challenged by observation showing that, for certain types of cancers, M1 macrophages can be associated with aggressive tumor growth and reduced survival (87, 88). In the case of skin cutaneous melanoma, high levels of M1 infiltrating macrophages are an indicator of better survival (89). Our results suggest a similar conclusion by showing that increased M1 signaling correlates with higher survival rate in this cancer type. Interestingly, we also observed in these patients a positive correlation between increased LV identity and improved survival. According to our results, we can speculate that in skin cutaneous melanoma, improved LV function and reduced lymph leakage favors a strong immune response by reducing M2 macrophage polarization condition with concomitant increase in M1 macrophages. Conversely, we found that increased M1 signature correlates with reduced survival in lung squamous cell carcinoma patients. M1/M2 signature in squamous cell lung cancer is still controversial, with some reports indicating that tumor islet-infiltrating M1 macrophages is a predictor of survival (90), while others show increase M1 macrophages at early but not late stages (91). Moreover, we observed that reduced LV signaling in lung squamous cell carcinoma patients showed better survival, which we can speculate that could be in part explained by a reduced M1 polarization process of intratumoral macrophages. Although in our study considered neither tumor stage nor macrophage localization, our results highlight that, depending on the cancer type analyzed, M1 macrophages can improve or decrease the survival of patients, and also underline that, depending on the cancer type, targeting LV function can be positive or detrimental to prognosis and survival. More studies are necessary to clarify these observations.

Some limitations of our work should be considered. First, even though we have previously shown that *Prox1^+/–^* mice have a defective LV and lymphatic leakage (14), we do not directly evaluate lymph leakage in the DSS or melanoma models in this mouse model. Second, although we showed that M2 macrophage polarization is promoted by lymph, the molecular mechanism involved in this phenomenon, or the potential mediators of this anti-inflammatory macrophage polarization remain elusive. However, because the different models used, either in the *Prox1^+/–^* mice and *WT* animals, together with the *in vivo* and *in vitro* results, we consider that this study provides enough evidence to explore the status of LV in different pathologies where macrophages play key roles, together to the potential use of therapeutic approaches to modulates LV function in order to promote or dampen an anti-inflammatory macrophage response.

In summary, our results show that *Prox1^+/–^* mice are less susceptible to intestinal inflammation with DSS treatment; lymph is capable of promoting an anti-inflammatory environment by modulating M2 macrophage polarization; lymph leakage reduces DSS-induced colitis but increased tumor growth; and intratumoral LV malfunction correlate with M1/M2 signature in different human cancers, impacting patients’ survival and suggesting that tumor cells, by an active process, could modulate intratumoral LV function to dampen antitumoral immune response, allowing tumor growth and dissemination through the body.

## Data Availability Statement

The original contributions presented in the study are included in the article/**Supplementary Material**. Further inquiries can be directed to the corresponding authors.

## Ethics Statement

The animal study was reviewed and approved by Institutional Animal Care and Use Committee at Universidad Autónoma de Chile.

## Author Contributions

AH and NE designed research. AH, AO-B, RL-A, and NE performed research. BH-R and GR performed bioinformatic analysis. AH, RL-A, AO-B, BH-R, GR, CL, and NE analyzed data and AH, AO-B, and NE wrote the paper. All authors contributed to the article and approved the submitted version.

## Funding

This work was funded by the following grants: FONDECYT de inicio N° 11190253 (AH), ANID - Millennium Science Initiative Program NCN19_168, FONDEQUIP EQM160063 and FONDECYT Regular N° 11140869 (GR), FONDECYT de inicio N° 11160592 and FONDECYT Regular N° 1201562 (NE), and ANID - Doctoral Fellowship N° 21201090 (BH-R). AH is a Latin American Fellow in the Biomedical Sciences, supported by The Pew Charitable Trusts.

## Conflict of Interest

The authors declare that the research was conducted in the absence of any commercial or financial relationships that could be construed as a potential conflict of interest.

## Publisher’s Note

All claims expressed in this article are solely those of the authors and do not necessarily represent those of their affiliated organizations, or those of the publisher, the editors and the reviewers. Any product that may be evaluated in this article, or claim that may be made by its manufacturer, is not guaranteed or endorsed by the publisher.
