# Supplementary material for: Lymph Leakage Promotes Immunosuppression by Enhancing Anti-Inflammatory Macrophage Polarization
Source: Front Immunol. 2022 May 19;13:841641. doi: 10.3389/fimmu.2022.841641 (PMC9160822; doi:10.3389/fimmu.2022.841641)
Supplement: Supplementary file 1 [file DataSheet_1.docx]

**Supplemental materials and methods**

**Resources:**

Expression levels and clinical data files from The Cancer Genome Atlas (TCGA) project were downloaded from the National Cancer Institute GDC PanCanAtlas project website (<https://gdc.cancer.gov/about-data/publications/pancanatlas>) (Liu et al., 2018). The type of cancer analyzed was melanoma (SKCM). The Macrophage and Lymphatic Vasculature (LV) gene signatures previously described in (Kowalczuk et al., 2018; Orecchioni et al., 2019), respectively, are shown with updated gene names, in Table 1. The .tsv file with gene expression levels, [EBPlusPlusAdjustPANCAN_IlluminaHiSeq_RNASeqV2.geneExp.tsv](http://api.gdc.cancer.gov/data/3586c0da-64d0-4b74-a449-5ff4d9136611) was downloaded from <http://api.gdc.cancer.gov/data/3586c0da-64d0-4b74-a449-5ff4d9136611/> and the excel file with TCGA-Clinical Data Resource (CDR) Outcomes ([TCGA-CDR-SupplementalTableS1.xlsx](https://api.gdc.cancer.gov/data/1b5f413e-a8d1-4d10-92eb-7c4ae739ed81)) was downloaded from <https://api.gdc.cancer.gov/data/1b5f413e-a8d1-4d10-92eb-7c4ae739ed81> (Liu et al., 2018). The .tsv file shows the expression of all the genes from different cell types belonging to more than two thousand patients, each suffering one of 33 types of cancer. The excel file shows among other information, the identifier (barcode) and survival information for the patients. In order to filter the gene expressions from particular cell types from all patients suffering one specific type of cancer, the barcodes were taken from the excel file and then used to filter the .tsv file. A Python script including Pandas and Numpy libraries was developed in order to filter and process the files. After obtaining the data, the methodology described in (Menares et al., 2019)[4] was used to correlate the expression of the gene signatures between two cell types: Macrophage (M1 and M2) and LV. In addition, we generated the survival Kaplan-Meier curves (K-M curves) for both cell types.

**Pearson correlation between Macrophage and LVS average z-score gene signature expression levels.**

In the correlogram charts, each dot or pair of coordinates (x; y) represents a patient, in which the x coordinate is the average of the z-scores of the genes in the gene signature of LV and, the y coordinate, is the average of z-scores of the genes in the gene signature of Macrophage (M1 or M2). The log-transformation of the expression level of each gene is log_10_ (gene expression level + 1), where the gene expression level comes from the .tsv file, which is in Upper Quartile FPKM. The z-score of the log-transformed expression of each gene was calculated using the average and standard deviation of the distribution of expressions for the same gene in the same cell type, across all patients with the same type of cancer. Then, was calculated the average of z-score per patient.

(<https://docs.gdc.cancer.gov/Data/Bioinformatics_Pipelines/Expression_mRNA_Pipeline/#fpkm>)

The correlation between the average z-score gene signature expression of Macrophages and LV was calculated using Pearson's correlation. The hypothesis test of significance of the correlation coefficient assesses if the calculated correlation is significantly different from zero.

**Survival or K-M curve**

The statistical analysis was performed in R. The survival data, in days, of the patients was extracted from the excel file, described in the previous section. The curves K-M were created using the “survival” and “survminer” R packages, according to the methodology in [4]. Patients with incomplete data were discarded (n_SKCM_ = 15 and n_LUSC_ = 3). In the survival curves, the patient's average z-scores of the gene signatures larger than the median, were grouped in light blue and average z-scores smaller than the median, were grouped in red. This way, K-M curves compare the survival of the patients with larger vs. smaller than median expression of genes in the gene signature of Macrophage cell types. The survival time in the x axis, in days, was recalculated in years, for better visualization.

Log rank hypothesis test was applied to both, light blue and red, K-M curves in order to see statistical differences between survival of the patients with larger vs. smaller than median levels of expression of the gene signature of Macrophage cell types.

**Heatmaps of gene expression of gene signature in patients with cancer types.**

The heatmaps were generated with “pheatmap” library in R using patients data presented in the K-M curves.

**Supplemental Table 2**

**Export_dataframe_SKCM.CSV:** File containing the information about the patients from TCGA clinical data and the mean z-scores used to generate the correlograms, and death/alive status to generate the K-M plots per cell type analysed in this work.

**Supplemental Figures**

**
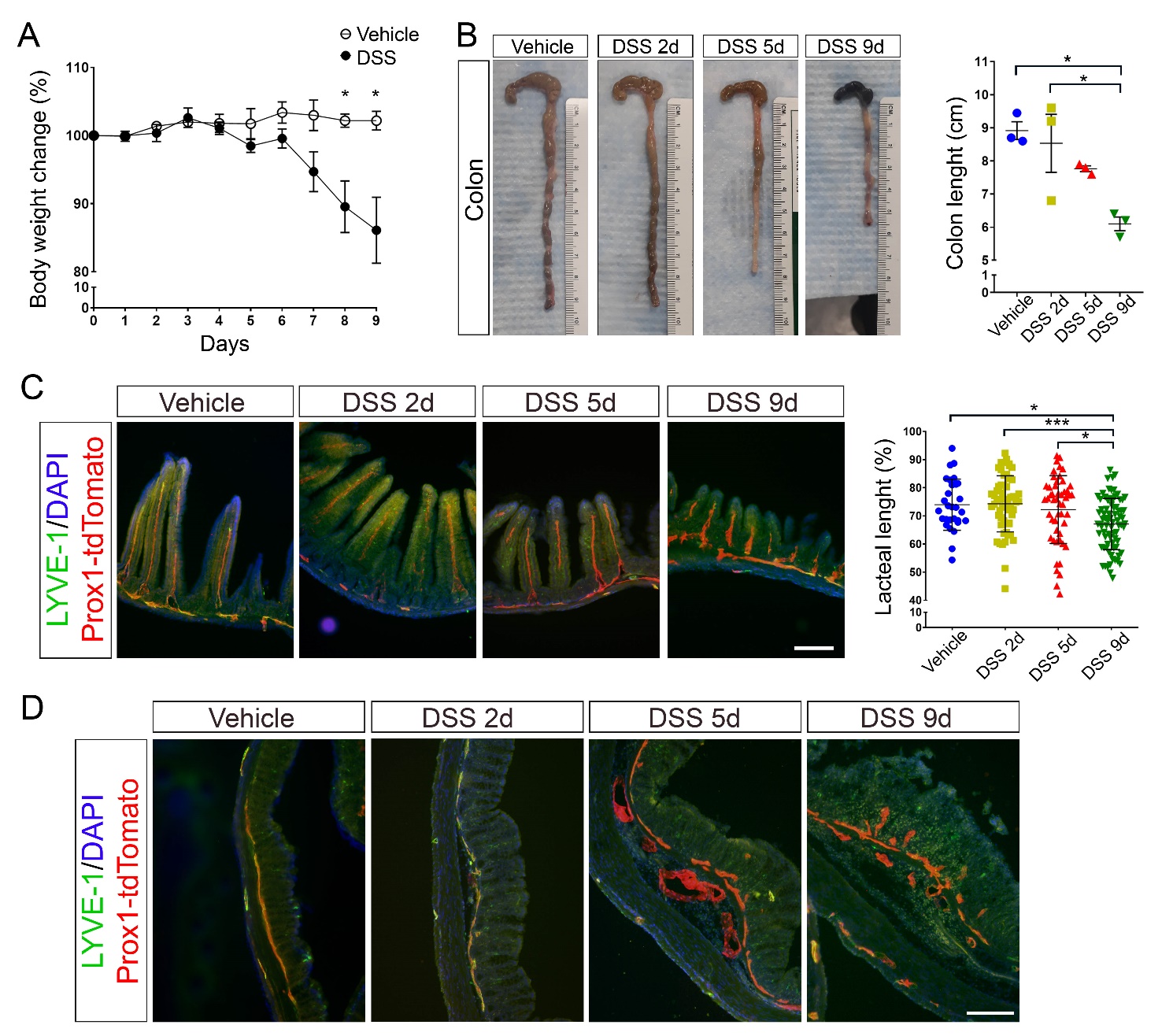
**

**Supplemental Figure 1. Lymphatic remodeling after DSS treatment in NMRI mice.** **(A)** Mouse body weight changes during DSS treatment. *WT* mice in NMRI background were treated with 3% (w/v) DSS or vehicle in the drinking water for 8 days and the weight was measured daily until day 9. Weight changes were calculated as a percentage of weight prior to DSS treatment (Day 0). **(B)** Macroscopic appearance (left) and quantification of the length of colons (right) from *WT* NMRI treated with 3% DSS or vehicle at different day after DSS treatment. **(C)** Representative images of lacteals (LYVE-1+; green and Prox1-tdTomato; red) on thick sections of the proximal duodenum from *WT* NMRI and *Prox1^+/-^* NMRI mice treated with vehicle or DSS for 9 days are shown (left). Scale bar is 200 µM**.** Quantification of relative lacteal length from the different groups is shown (right). Dots indicate values of 29–78 villi/group in n = 10 mice/group pooled from two independent experiments. **(D)** The colon lymphatics were stained using LYVE-1 (Green) in Prox1-tdTomato NMRI mice (Red). LYVE-1+ Prox1+ indicates the lymphatic vessels. Scale bar is 200 µM. Data pooled from 2 (n = 10 mice per group) independent experiments is shown. Data are plotted as means ± SEM. * p < 0.05; *** p < 0.001 by 1-way ANOVA with post hoc Tukey's multiple comparisons test.

**
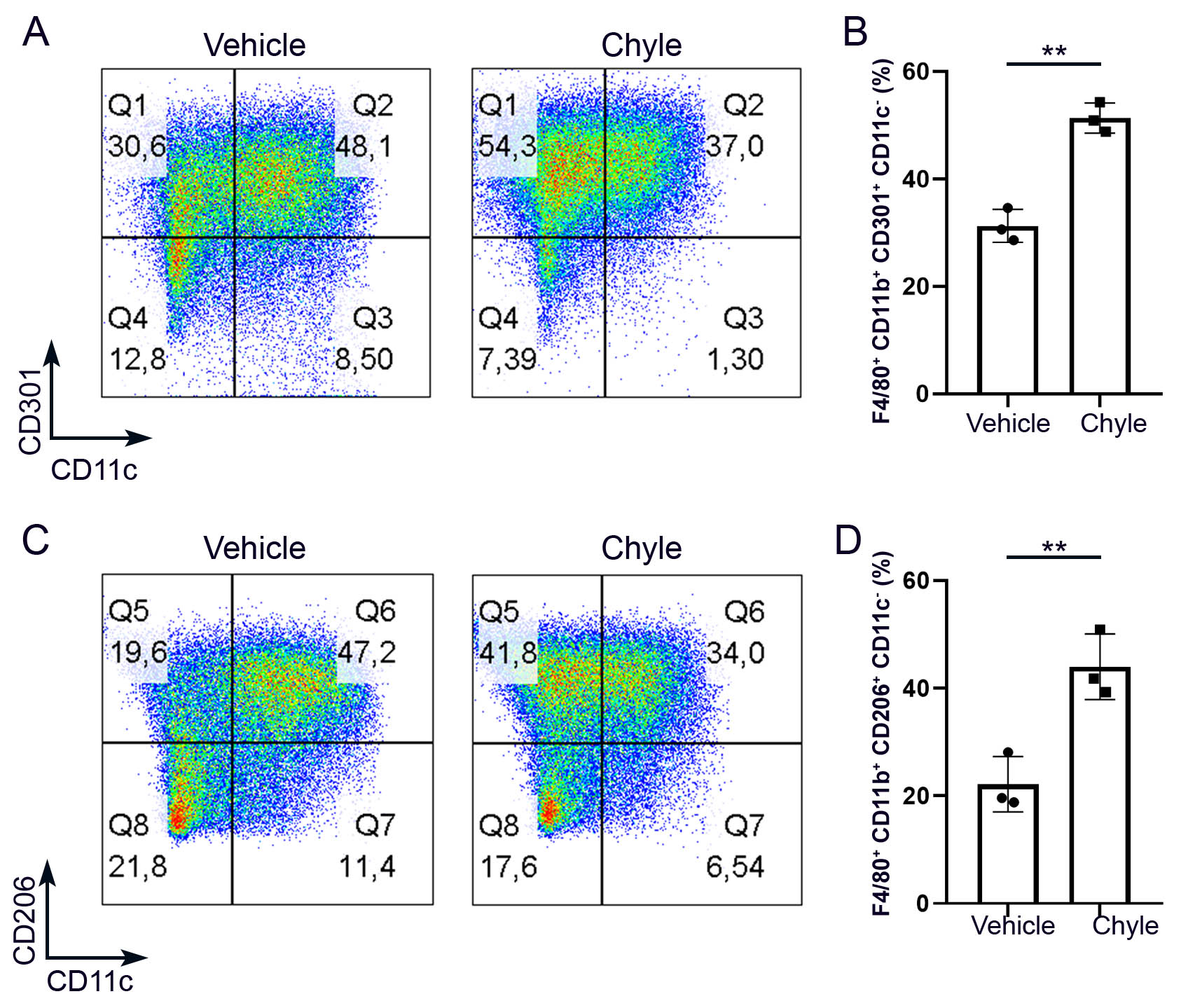
**

**Supplemental Figure 2. Enhanced M2 polarization of Bone-marrow-derived macrophage (BMDMs) in response to chyle.** BMDMs were obtained from *WT* mice and cultured under M2 polarization condition with or without 2 µL of chyle or vehicle for 24 hrs. M2 macrophages were defined as F4/80^+^CD11b^+^CD301^+^CD11c^-^ (A and B) or F4/80^+^CD11b^+^CD206^+^CD11c^-^ (C and D) by FACS. Representative density plots (A and C) and quantification of 3 independent experiments (B and D) are shown. Error bars indicate SEM. ** p < 0.01 by Unpaired t-test


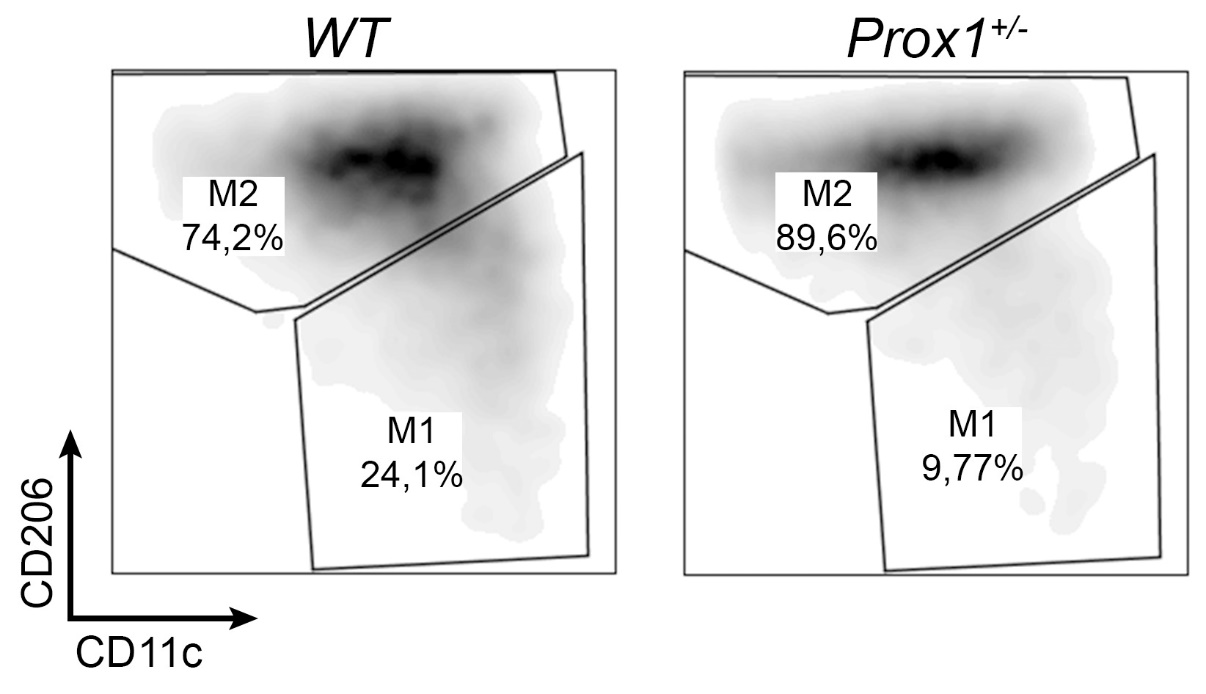


**Supplemental Figure 3. Increased M2 macrophages in stromal vascular fraction (SVF) of *Prox1^+/-^* mice**. Flow cytometry analysis of F4/80^+^CD11b^+^CD11c^+^CD206^-^ (M1) and F4/80^+^CD11b^+^CD11c^-^CD206^+^ (M2) macrophages in the SVF of visceral adipose tissue obtained from 5-month-old *WT* and *Prox1^+/-^* mice are shown. Density plots are representative from 3 mice per group.

**
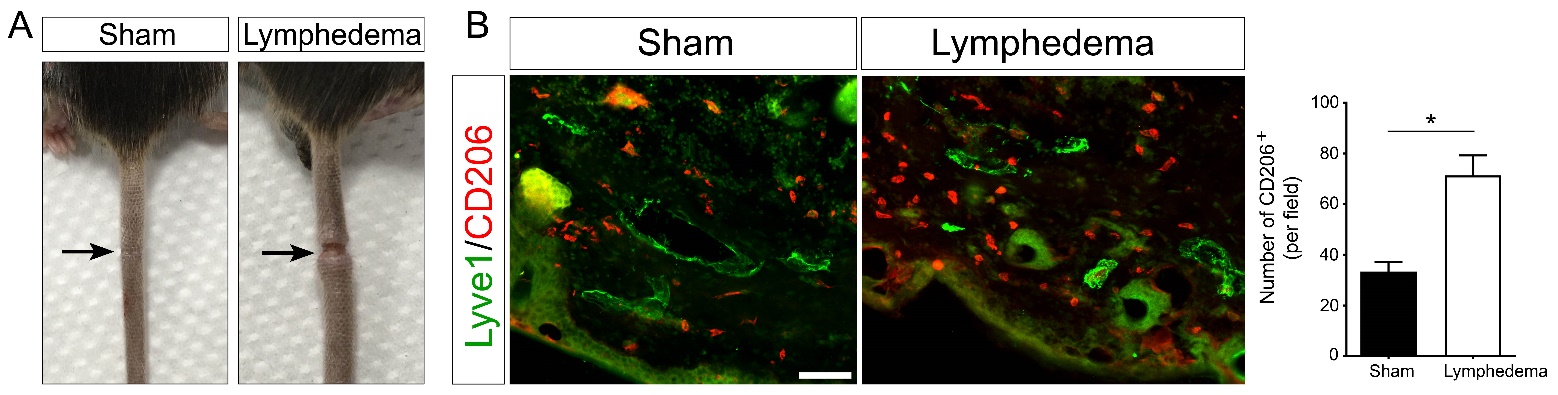
**

**Supplemental Figure 4. Increased numbers of M2 macrophages in the lymphedema tissue at early days postsurgery**. Mouse tail model of acquired lymphedema was surgically induced by thermal ablation of lymphatic trunks (Lymphedema). Skin incision without lymphatic ablation surgery was performed in control group (Sham). **(A)**. Representative tails in the Sham and Lymphedema group indicating in the black arrow the incision site on postoperative days 4. **(B)** CD206+ cells in the lymphedematous tissue of Sham and Lymphedema group were analyzed by immunofluorescence staining. Representative images (left) and quantification (right) are shown. Scale bar is 100 µM. Data pooled from 2 (n = 6 mice per group) independent experiments is shown. Error bars indicate SEM. * p < 0.05 by Unpaired t-test.


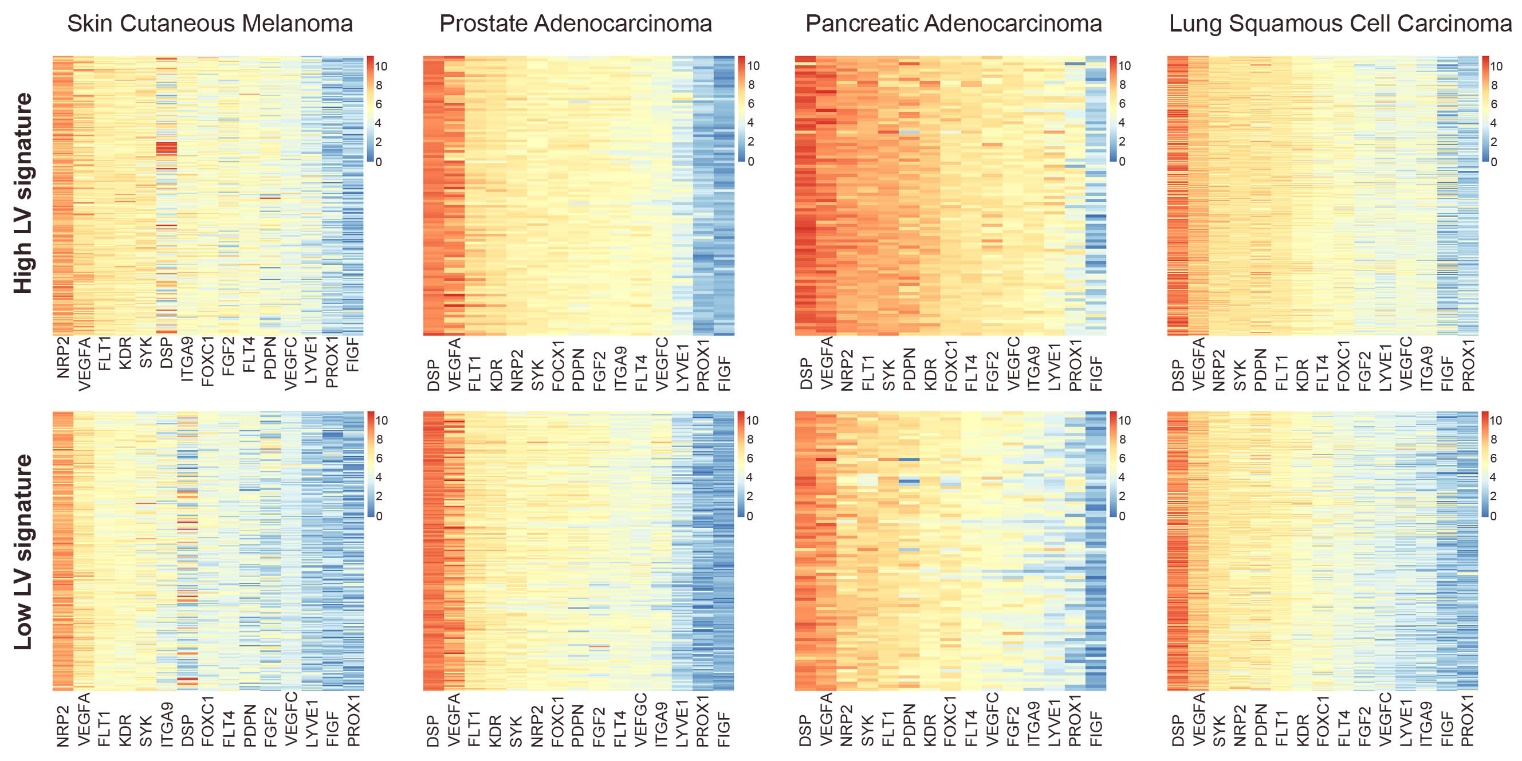


**Supplemental Figure 5. Heatmaps of the normalized expression of genes from the Lymphatic Vasculature gene signature in different cancer types.** The types of cancer are: Skin Cutaneous Melanoma, Prostate Adenocarcinoma, Pancreatic Adenocarcinoma or Lung squamous cell carcinoma.Each of the heatmaps refers to a gene signature associated to the lymphatic vasculature (LV). Therefore, in each heatmap, each column refers to a gene of the gene signature. Each line of a heatmap refers to a patient with a type of cancer. The cells of a heatmap represent the expression of the gene and patient. To enhance the differences between the expression in the higher than mean z-score patients (High LV signature, upper panels) and the lower than mean z-score patients (Low LV signature, lower panels), the gene expression, in FPKM (Fragments Per Kilobase Million), was scaled using natural logarithm (ln).The values range from lowest ln(gene expression) equals 1 (in blue) to the largest ln(gene expression) equals 10 (in red). The genes of each signature and heatmap were sorted from the most expressed (leftmost column of a heatmap) to the less expressed gene (rightmost column).

| **Gene Signatures** | | | | |
| --- | --- | --- | --- | --- |
| **Macrophages** | | | | **Lymphatic Vasculature** |
| **M1 Classic** | **M1 No Classic** | **M2 Classic** | **M2 No Classic** | **LVs** |
| AFF1 | ARL5B | AAK1 | AFF1 | DSP |
| BRAF | BBS4 | ACO2 | CBX5 | FGF2 |
| CLN5 | CENPJ | CBX5 | CLEC5A | FIGF |
| CXCL16 | CXCL16 | CDC23 | EIF4E3 | FLT1 |
| DDX27 | HERC3 | COMMD6 | GSPT1 | FLT4 |
| EIF4E3 | HIBCH | CTNNBIP1 | NNT | FOXC1 |
| GSPT1 | HOXB4 | FAHD1 | PLEKHF1 | ITGA9 |
| PLEKHF1 | PCGF5 | HIBCH | POLR3K | KDR |
| RNF11 | PPP3CB | HMGCL | PPP2R1B | LYVE1 |
| SEC23B | PSEN1 | LNX2 | RPS15 | NRP2 |
| USP25 | RNF11 | MDH2 | SLC1A5 | PDPN |
|  | SEC23B | MRPS35 | SLC7A1 | PROX1 |
|  | USP25 | NFS1 | SULT1A1 | SYK |
|  |  | ORMDL1 | WDR4 | VEGFA |
|  |  | PHB2 | ZWILCH | VEGFC |
|  |  | POLR3K |  |  |
|  |  | SELPLG |  |  |
|  |  | STUB1 |  |  |
|  |  | SUMF1 |  |  |
|  |  | TINF2 |  |  |
|  |  | XRCC6 |  |  |
|  |  |  |  |  |

**Supplemental Table 1. Table of genes that define gene signatures**. The table shows the genes that conform the gene signature in: Macrophage M1 Classic; Macrophage M1 No Classic; Macrophage M2 Classic; Macrophage M2 No Classic; LV = Lymphatic Vasculature. The gene names were updated according to NCBI’s Gene Database.

**References**

Menares, E., Gálvez-Cancino, F., Cáceres-Morgado, P., Ghorani, E., López, E., Díaz, X., Saavedra-Almarza, J., Figueroa, D. A., Roa, E., Quezada, S. A., et al. (2019). Tissue-resident memory CD8+ T cells amplify anti-tumor immunity by triggering antigen spreading through dendritic cells. Nat commun. 10, 4401.

Kowalczuk, O., Laudanski, J., Laudanski, W., Niklinska, W.E., Kozlowski, M., Niklinski, J. (2018). Lymphatics-associated genes are downregulated at transcription level in non-small cell lung cancer. Oncol Lett 15, 6752–6762.

Liu, J., Lichtenberg, T., Hoadley, K.A., Poisson, L.M., Lazar, A.J., Cherniack, A.D., Kovatich, A.J., Benz, C.C., Levine, D.A., Lee, A. v., Omberg, L., et al. (2018). An Integrated TCGA Pan-Cancer Clinical Data Resource to Drive High-Quality Survival Outcome Analytics. Cell. 173, 400-416.e11.

Orecchioni, M., Ghosheh, Y., Pramod, A.B., Ley, K. (2019). Macrophage polarization: Different gene signatures in M1(Lps+) vs. Classically and M2(LPS-) vs. Alternatively activated macrophages. Front Immunol. 10, 1084.
